# Studies of Black Diamond as an antibacterial surface for Gram Negative bacteria: the interplay between chemical and mechanical bactericidal activity

**DOI:** 10.1038/s41598-019-45280-2

**Published:** 2019-06-19

**Authors:** O. Dunseath, E. J. W. Smith, T. Al-Jeda, J. A. Smith, S. King, P. W. May, A. H. Nobbs, G. Hazell, C. C. Welch, B. Su

**Affiliations:** 1 School of Chemistry, University of Bristol, Bristol, BS8 1TS United Kingdom; 2 Bristol Dental School, University of Bristol, Lower Maudlin Street, Bristol, BS1 2LY United Kingdom; 3 Oxford Instruments Plasma Technology, Yatton Bristol, BS49 4AP United Kingdom

**Keywords:** Biological techniques, Biomaterials

## Abstract

‘Black silicon’ (bSi) samples with surfaces covered in nanoneedles of length ~5 µm were fabricated using a plasma etching process and then coated with a conformal uniform layer of diamond using hot filament chemical vapour deposition to produce ‘black diamond’ (bD) nanostructures. The diamond needles were then chemically terminated with H, O, NH_2_ or F using plasma treatment, and the hydrophilicity of the resulting surfaces were assessed using water droplet contact-angle measurements, and scaled in the order O > H ≈NH_2_ >F, with the F-terminated surface being superhydrophobic. The effectiveness of these differently terminated bD needles in killing the Gram-negative bacterium *E. coli* was semi-quantified by Live/Dead staining and fluorescence microscopy, and visualised by environmental scanning electron microscopy. The total number of adhered bacteria was consistent for all the nanostructured bD surfaces at around 50% of the value for the flat diamond control. This, combined with a chemical bactericidal effect of 20–30%, shows that the nanostructured bD surfaces supported significantly fewer viable *E. coli* than flat surfaces. Moreover, the bD surfaces were particularly effective at preventing the establishment of bacterial aggregates – a precursor to biofilm formation. The percentage of dead bacteria also decreased as a function of hydrophilicity. These results are consistent with a predominantly mechanical mechanism for bacteria death based on the stretching and disruption of the cell membrane, combined with an additional effect from the chemical nature of the surface.

## Introduction

Due to the extreme properties of diamond together with their growing availability and affordability from a number of commercial suppliers, chemical vapour deposition (CVD) diamond films^1^ are beginning to find an increasing number of applications in material science. Structuring the diamond surface on the micro- or nanoscale can greatly increase the effective surface area, leading to applications as electrochemical electrodes with high sensitivity, selectivity, and capacitance values^2,3^. Our group recently reported^4^ a fabrication method for high-surface-area boron-doped diamond electrodes using ‘black silicon’ (bSi) as a template. Black Si is a nanostructured material composed of high-aspect-ratio nanospikes or nanoneedles on its surface that are produced through plasma etching of a Si wafer. The formation mechanism is believed to be due to particulates from the chamber sidewall, or ones that have homogeneously nucleated in the gas phase, that deposit onto the Si wafer surface and act as micromasks. A highly anisotropic etch process then etches the Si into high-aspect-ratio needles. The name ‘black silicon’ originates from its deep black colour, resulting from the absorption of >99% of the visible light falling onto its surface. Black Si has now found applications in photovoltaics^5,6^ and in biomedical sensing^7,8^.

We recently reported^4,9^ that the spiky diamond-coated bSi surface, which we called ‘black diamond’ (bD), generates a mechanical bactericidal effect, killing the Gram-negative bacteria *Pseudomonas aeruginosa* and *Escherichia coli* at high rates. The uncoated bSi needles also act as a bactericidal surface^10^, but the nanostructured bSi surface is delicate and easily damaged – even a human fingernail dragged across the surface can break and/or dislodge the needles. The advantage of the diamond coating is that the structures become more robust and less likely to become damaged. In this case, no obvious damage to the bD needles was caused by the fingernail test, however more quantitative tests have yet to be performed. Although some studies have reported on the bactericidal efficacy of other carbon nanostructured surfaces, such as carbon nanotubes, and graphene^11^, and of flat nanodiamond^12^, our previous report^9^ was the first in which the antimicrobial properties of bD nanoneedle surfaces were investigated systematically in terms of needle length, tip diameter and needle density. The length of the needles was found to be less important than their separation, *i.e*. areal density. This is consistent with reports of bactericidal activity on other synthetic^13–16^ and natural^17^ nanostructured materials, and provides evidence for a model for mechanical-induced bacteria death based on the stretching and disruption of the cell membrane^18–21^. Here, high-aspect-ratio nanofeatures have been shown to control microbial growth in a surface-chemistry-independent manner with a physico-mechanical mechanism. Such bactericidal effects are due to the presence of nanostructures which are able to either penetrate the bacterial cell wall upon contact or rupture the cell wall by placing it under mechanical stress.

Because the mechanism for bacterial death is believed to be predominantly mechanical, (bio)chemical effects, such as the interaction of the bacteria with the chemical species attached to the surface of the substrate are often overlooked. Bacteria tend to adhere strongly to surfaces which are hydrophilic but do not adhere as well on hydrophobic surfaces^22^. Diamond is particularly amenable to study these effects because its surface termination can be readily modified by simple chemical treatment or exposure to a reactive plasma. As-deposited CVD diamond is hydrogen terminated, which makes the surface slightly hydrophobic. Oxidising the surface, such that it is covered in hydroxyl (OH) or O groups, either as bridging ethers (‒O‒), ketones (>C=O), carboxylic acids (‒COOH) or lactones (‒COO‒), makes the surface hydrophilic, whereas fluorinating the surface makes it extremely hydrophobic. The effect of these chemically modified flat diamond surfaces on the adhesion of *E. coli* was studied by Budil *et al*.^23^. They found that in a water-based (mineral M9) medium, the H- and F-terminated diamond films reduced bacterial adhesion by ~50%, whereas in a more complex medium (Luria-Bertani) there was no reduction in bacterial adhesion. These differences were attributed to the passivation of the H- or F-terminated diamond films by organic molecules adsorbed from the complex medium. In contrast, for O-terminated diamond films, no anti-adhesive effect was observed in either cultivation medium. For the purposes of anti-bacterial surfaces, these results beg the question: is it better to prevent the bacteria from sticking to the surface, or to encourage them to adhere but then efficiently kill them before they can form colonies/biofilms? In this paper we have investigated this by studying the viability of *E. coli* on F-, O-, H- and NH_2_-terminated bD needles, in order to determine whether the chemical effects of the surface termination or the mechanical effects of the nanostructures are more significant in bactericidal activity. To our knowledge, this is the first time these two effects have been directly compared using the same substrate type and material.

Previous work has shown that some nanostructures exhibit a greater bactericidal effect against Gram-negative bacteria than Gram-positive bacteria^9,24^. It is suggested that this is due to the difference in thickness of the bacterial cell wall. For these reasons, *E. coli* is often chosen as the model bacterium with which to test bactericidal efficacy. *E. coli* is a motile, rod-shaped Gram-negative bacterium of size ~2 μm by ~0.5 μm that is commonly isolated from nosocomial infections^25^. *E. coli* expresses surface-bound organelles known as *fimbriae*, or attachment *pili*, which are hair-like proteinaceous appendages used to facilitate cell attachment to surfaces. During biofilm formation, *E. coli* also produces extracellular polymeric substance (EPS), and adhesive material that is used to anchor cells to a surface and withstand shear forces.

Infection by bacteria such as *E. coli* becomes particularly problematic when they adhere to a suitable surface, grow, multiply, and form a mature biofilm. Bacterial biofilms are populations of bacteria cells that assemble into organised structures at an interface, and become embedded in a protective EPS matrix^26^. Once a mature biofilm has formed, the bacteria can become 10–1,000-fold less susceptible to attack from antimicrobial agents than the same bacteria grown in free-floating (planktonic) culture^27^. This is because the effect of antibiotic treatment is largely limited to the outer layer of the biofilm^28,29^ and the reagents cannot penetrate to the bacteria at the centre of the colony. Biofilms account for over 80% of microbial infections in the body^30^, and are commonly associated with diseases such as colitis, conjunctivitis and gingivitis. Because of its tendency to form biofilms, *E. coli* is a frequent coloniser of medical devices such as catheters^31^, and is thus a primary cause of urogenital infections^32^. In this paper, *E. coli* is the model bacterium used for the tests, and the length and density of the nanoneedles on the bD surface was optimised for this bacterium based on previous work^9^.

## Experimental

### Black silicon fabrication

**Figure 1:**
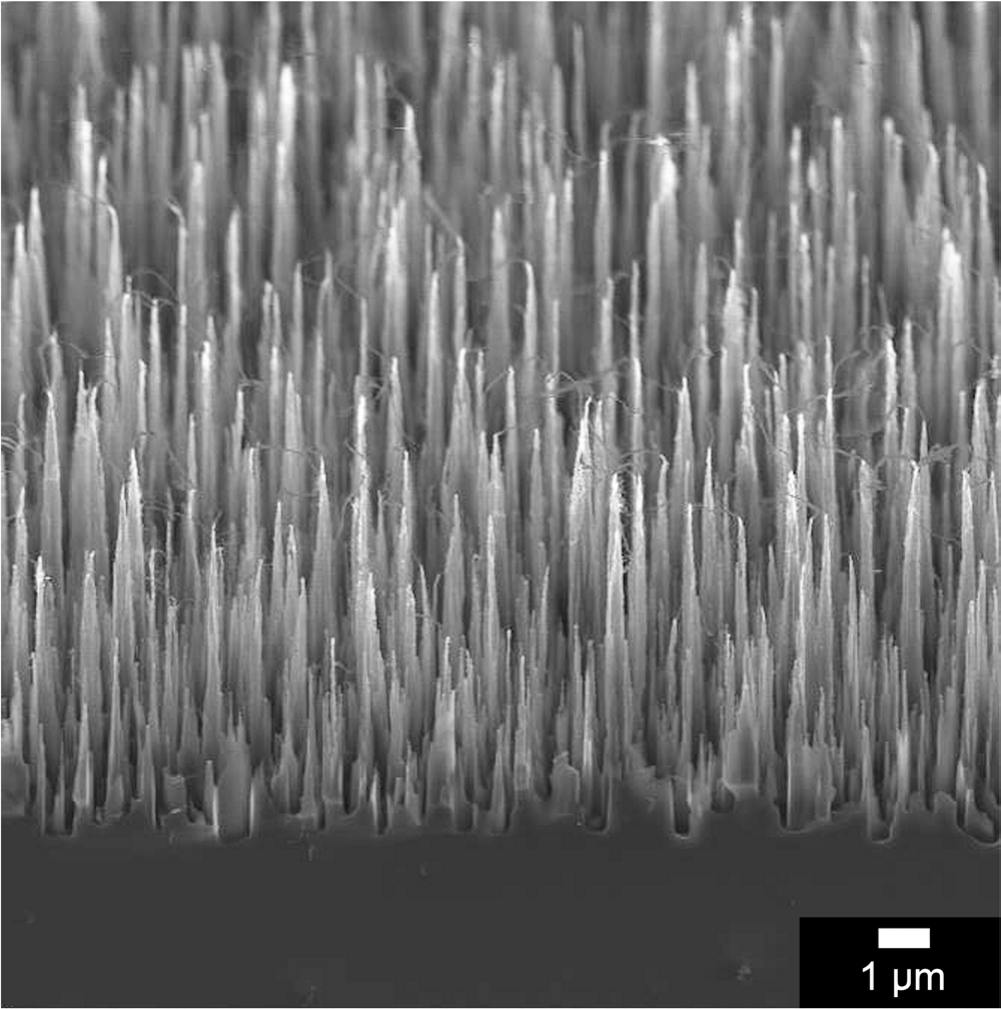
Scanning electron microscope (SEM) image of the bSi needles showing the needle profile and variation in height.

The bSi substrates were prepared by plasma etching of n-doped single-crystal silicon (100) wafers in an Oxford Instruments System 133 reactive ion etching (RIE) system fitted with a ICP380 inductively coupled plasma (ICP) source^33^. This reactor uses inductively coupled radio frequency (RF) power to sustain a plasma, and a second RF power supply capacitively coupled to one electrode (as in a standard RIE system) to induce a DC bias which controls the ion bombardment energy onto the substrate sitting on that electrode. The etch gas was a mixture of Cl_2_ (48 sccm) and O_2_ (2 sccm) at 15 mtorr pressure for 10 min. The wafer temperature was maintained at 20 °C by flowing He coolant gas at 10 torr under the backside of the wafer. The RIE and ICP powers were 100 W and 600 W, respectively, with a DC bias of −227 V. This process produced a bSi wafer as detailed in Table 1 and shown in Fig. 1. The wafer was then cleaved into multiple identical square samples ~1 cm^2^ in size, suitable for subsequent testing.

**Table 1:** Details of the bSi and bD needles as measured by scanning electron microscopy.

Sample	Tip radius/nm	Length/µm	Needle areal density/µm^−2^
bSi	30 ± 5	4.6 ± 1.5	1.4
bD	300 ± 50	3.0 ± 1.3	1.2

### Diamond deposition

The bSi samples were seeded using a suspension of ~4–10 nm detonation nanodiamond (DND) in methanol using an electrospray process^34^. The DND suspension was placed under high potential difference (35 kV) with respect to the grounded sample, and due to electrostatic attraction, the suspension sprayed onto the sample and coated all the surfaces, including the vertical sides of the needles, with a near monolayer of DND seeds. The seeded samples were then placed into a hot filament CVD (HFCVD) reactor where ~0.3 µm of microcrystalline diamond (MCD) was deposited using standard CVD conditions^1^: 20 torr pressure, Ta filament at 2100 °C, substrate temperature ~900 °C, 1%CH_4_/H_2_ gas mixture. A growth time of ~60 min allowed a continuous and conformal diamond coating to be deposited without filling in the gaps between the needles. The methane supply was turned off for the last 1 min of growth, and the samples were cooled down under hydrogen. This was to ensure that the diamond surface was fully hydrogen terminated.

**Figure 2:**
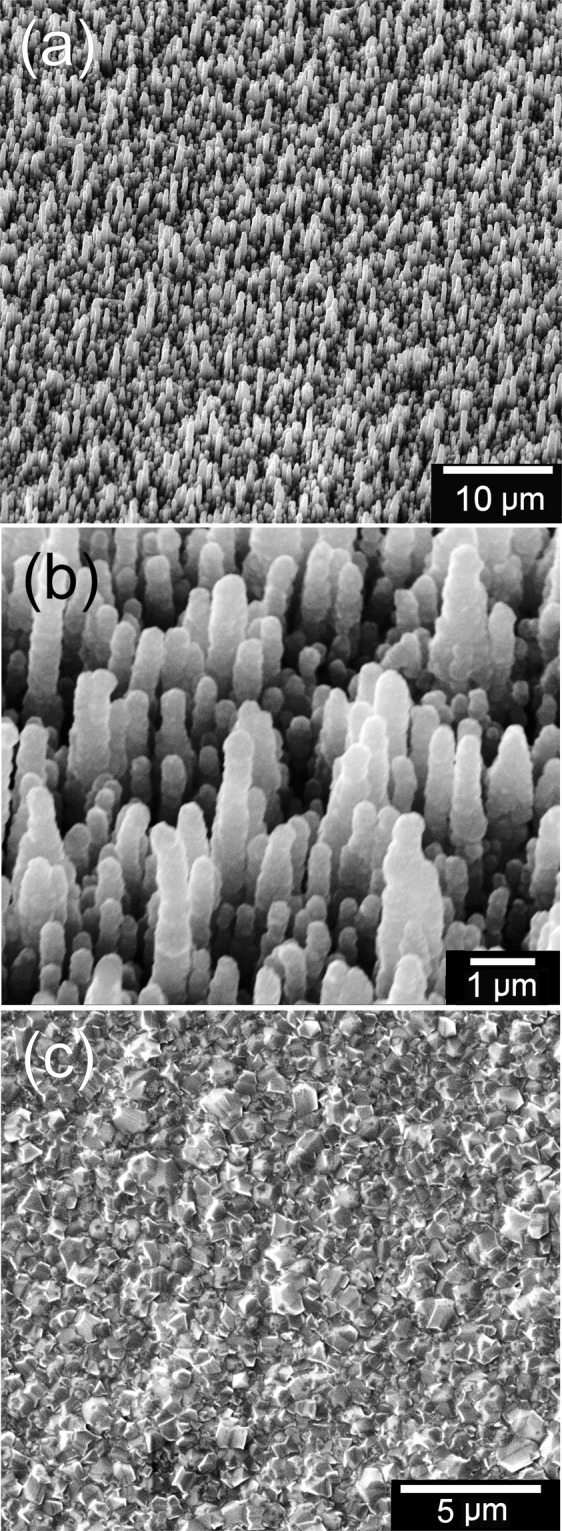
SEM images of the bD samples at (**a**) low and (**b**) high magnification. (**c**) The ‘flat’ diamond control sample.

The resulting bD samples remained deep black due to absorption of visible light, and their microstructure can be seen in Fig. 2(a),(b). Laser Raman analysis of the deposited coating confirms it is consistent with small-grained CVD diamond (see Supplementary Fig. S1). Due to the submicron thickness of the diamond layer, the diamond grain size is small (<100 nm) so the MCD film appears rounded with only small facets visible. The bSi needles were coated uniformly with diamond to a thickness of ~0.3 μm, leaving a surface which was rounded compared to the uncoated Si needles (Fig. 2(a,b)). After diamond coating, the density of the needles remained roughly unchanged, while the average length decreased by ~1.5 μm (presumably due to etching back of the ultra-sharp Si needle points), and the tip became significantly less sharp, with the tip radius increasing from ~30 nm to ~300 nm (see Table 1).

Control samples of diamond films grown on flat (100) Si wafers were deposited following the same seeding and growth conditions, except for extension of the growth duration to 8 h to ensure a continuous polycrystalline diamond coating ~3 μm thick. Although we shall refer to these control samples as ‘flat’ diamond, it should be noted that the surface is faceted on a micron scale from the various randomly oriented diamond crystallites, which gives an overall surface roughness of about 0.2 μm, as seen in Fig. 2(c).

### Modifying the diamond surface termination

To alter the surface chemistry, identical bD samples were exposed to plasmas of three different gases: O_2_, NH_3_ and SF_6_, using a modified 2.1 kV a.c. plasma sputter-coater (Edwards S150A) apparatus. The optimal process conditions for obtaining the maximum surface coverage of the three adsorbates on flat diamond substrates had been previously determined^35^ (10 sccm of gas flowing, 0.1–1 torr pressure, substrate grounded and maintained at room temperature) and typically required exposure to the relevant plasma for between 10–30 s. Shorter plasma exposure times gave less coverage, whilst longer times often led to unwanted etching of the diamond, particularly when using O_2_ or SF_6_ gases. X-ray photoelectron spectroscopy (XPS) was used to confirm and quantify the presence of O and N on previous flat diamond surfaces after plasma treatment^35^. Results showed that after O_2_ plasma treatment, O was present at or above one monolayer coverage, whilst after NH_3_ treatment the coverage of N was approximately one monolayer. In either case, however, it was not possible to distinguish between different chemical moieties (*e.g*. between N, NH, or NH_2_, or the ether, ketone or lactone O terminations). The coverage of F was not measured after SF_6_ plasma exposure, but recent experiments on a similar sample suggests it was also between 0.5–1 monolayer.

These XPS measurements were made on flat diamond control samples – the actual values for coverage on the nanostructured bD samples were not measured, and may differ from these values. Therefore, to obtain a separate semi-quantitative measure of the degree of surface coverage and relative hydrophilicity, the 4 different types of bD sample were analysed using water droplet contact angle (CA) measurements. Samples were washed with ethanol and air dried before testing using a Krüss droplet shape analyser in combination with *Advance* software. 5 µL droplets were pipetted onto each surface whilst the software recorded the CA at 1 s intervals until the value stabilised.

### Bactericidal surface testing

#### Bacterial culture preparation

Broth cultures of *E. coli* strain DH5-α were grown in Tryptic Soy Broth (TSB, Oxoid) under aerobic conditions at 37 °C for 16 h. The suspension was sub-cultured into TSB and grown to mid-exponential phase (OD_600_ = 0.5), upon which the bacterial cells were harvested by centrifugation (7 min, 5000 *g*) and washed with Tris-HCl buffer (10 mM, pH 7), before resuspending in buffer to OD_600_ = 0.3 (equivalent to ~10^7^ CFU/mL).

#### Bacterial adhesion assay

All surfaces to be treated with *E. coli*, including the bD samples, were washed with 70% ethanol and air dried, before submerging in the bacterial suspension (2 mL) in a 12-well microtitre plate and incubating under static conditions (37 °C, 1 h). After incubation, surfaces were rinsed with Tris-HCl buffer (3×) to remove any non-adhered cells. BacLight Live/Dead viability stain was applied to the samples according to manufacturer’s instructions, and incubated for 15 min at 21 °C in darkness. Finally, samples were rinsed with buffer before visualising the adherent bacteria using a fluorescence microscope (Leica). To determine levels of bacterial viability, 4 images of each surface were taken (magnification ×20, each corresponding to an area of 0.097 mm²), and numbers of live (SYTO9, green) and dead (propidium iodide, red) cells were recorded using *ImageJ* software.

#### Scanning electron microscopy (SEM)

On completion of Live/Dead assays, bacteria were fixed on the samples overnight using glutaraldehyde (2.5%) at 4 °C. Samples were then washed in the same buffer (0.1 M) and dehydrated using a graded ethanol series (20%, 50%, 70%, 90%, 100%; 10 min each), followed by critical-point drying, and Au-sputter coating before observation in an environmental SEM.

## Results

### Contact angle measurements

**Figure 3:**
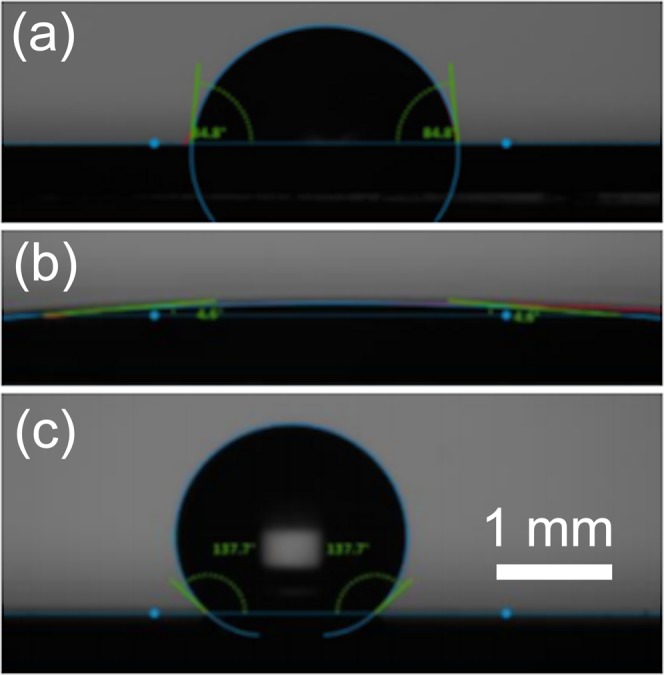
Representative images of the water droplet profiles used to measure the CAs for (**a**) H-terminated, (**b**) O-terminated, and (**c**) F-terminated bD.

**Figure 4:**
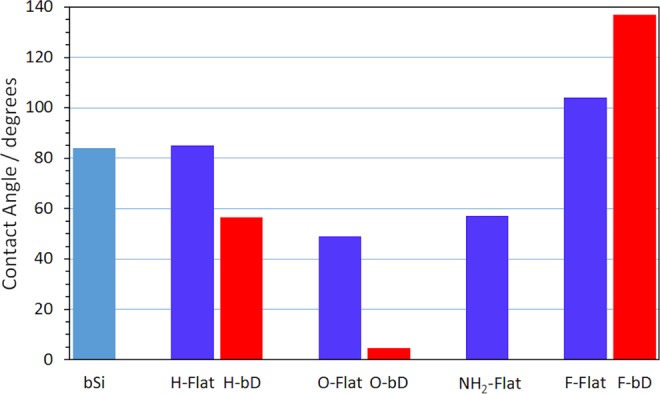
Bar chart showing the water droplet CA measured at equilibrium on the differently terminated flat (blue bars) or nanostructured bSi (light blue bar)/bD (red bars) surfaces.

Representative images from the CA tests are shown in Fig. 3, and the values given in Fig. 4. The CA of the H-terminated flat control diamond film was recorded as 84.8°, in agreement with previously reported literature values^36^, with uncoated bSi exhibiting a very similar CA of 83.8°. The CA values for O-, N- and F-terminated flat diamond are also in line with those reported previously. However, unlike the flat samples, the interaction of water with both the bSi and bD surfaces was found to be a dynamic process, with the CA steadily decreasing over time as the liquid spread into the nanostructure. Thus, CA measurements of these nanostructured materials were delayed until after the droplet size/shape had reached equilibrium, typically 30 s. The CA values for the bD samples follow the same trend as that of the flat diamond samples, although the absolute values of each are somewhat different as a result of the effect of the nanostructure. Nevertheless, it is clear that with a CA of 4.6° the O-terminated bD is very hydrophilic, while conversely, with a CA of 137.0°, the F-terminated bD surface can be considered super-hydrophobic. The H- and NH_2_-terminated surfaces are both moderately hydrophobic surfaces. We can thus scale the hydrophilicity in the order O > H ≈NH_2_ > F.

### Bacterial adhesion

**Figure 5:**
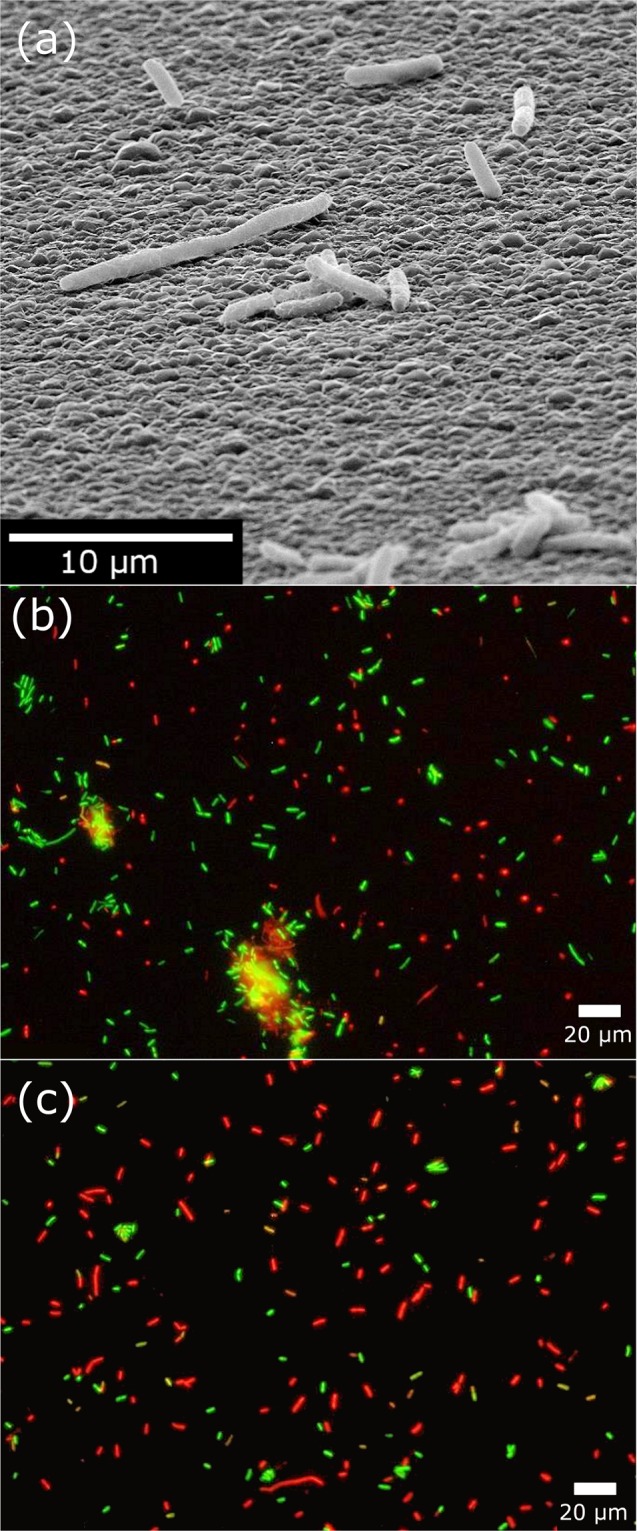
(**a**) SEM image of bacterial cells adhered to the flat diamond sample showing instances of cell aggregation. Representative fluorescence micrographs of (**b**) flat diamond thin film compared to (**c**) bD, showing the reduction in cell aggregates on the nanostructures. Live bacterial cells were stained green; red staining indicates dead cells.

A qualitative observation made from the fluorescence microscopy images was the reduction in the numbers of bacterial aggregates, a precursor to biofilm formation on the nanostructured surfaces. The relative number of clusters of live bacteria cells, as visualised by SEM in Fig. 5(a), can be compared in Fig. 5(b),(c) for the flat and nanostructured diamond surfaces, respectively. These aggregates appeared more frequently on the flat diamond control than on the needle-coated surfaces. This was attributed to the fact that physical restriction of bacterial cells bound to the discontinuous horizontal surface of the nanostructured substratum relative to the flat surface impaired bacterial movement and hindered biofilm formation.

### Effect of surface termination on bacterial viability

**Figure 6:**
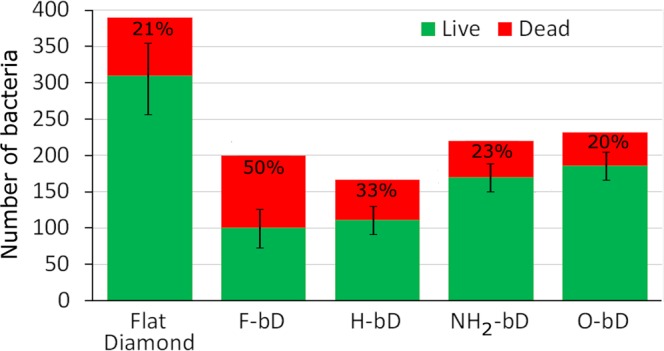
Bacterial viability on surfaces with the different terminations, compared to that of the H-terminated flat diamond control. Numbers of live (green) and dead (red) cells are indicated, together with the corresponding percentage of dead cells. The uncertainty in the measurements are shown as error bars representing one standard deviation of the number of dead cells based upon 4 repeats of the experiment.

The results of the Live/Dead experiments on the various samples can be seen in Fig. 6. The total number of adhered bacteria remained consistent for all the nanostructured bD surfaces at around 50% of the value for the H-terminated flat diamond control. Comparing the values for the H-terminated flat diamond with those for the H-terminated bD surface suggests that nanostructuring the surface directly impairs bacterial adhesion by a factor of around 2. Measurements for O-, F- and NH_2_-terminated flat diamond were not made, so no direct comparisons can be stated for flat versus nanostructured surfaces for these other terminations, although we assume that the magnitude of the biocidal affect would be similar to that seen for the H-terminated surfaces.

To make it easier to visualise trends for the nanostructured films, the data in Fig. 6 have been ordered in terms of increasing hydrophilicity. Looking at the H, NH_2_, and O samples first, the total number of bacteria that adhered increased a small amount with increasing hydrophilicity. This is unsurprising, however the trend is not as strong as might be expected. The percentage of dead bacteria decreased slightly as a function of hydrophilicity, showing that although more bacteria were sticking to the hydrophilic surfaces, relatively fewer of them were being killed.

In contrast, the superhydrophobic F-terminated surface behaved somewhat differently than the other surfaces. Surprisingly, for a surface that is supposed to repel bacteria, the number of adhered bacteria was similar to that on the hydrophilic surfaces. However, the percentage killed was nearly double that on the other bD surfaces. This implies that surface termination has minimal impact on the capacity for bacteria to adhere, but that hydrophobic surfaces have a greater bactericidal effect.

### SEM visualisation

**Figure 7:**
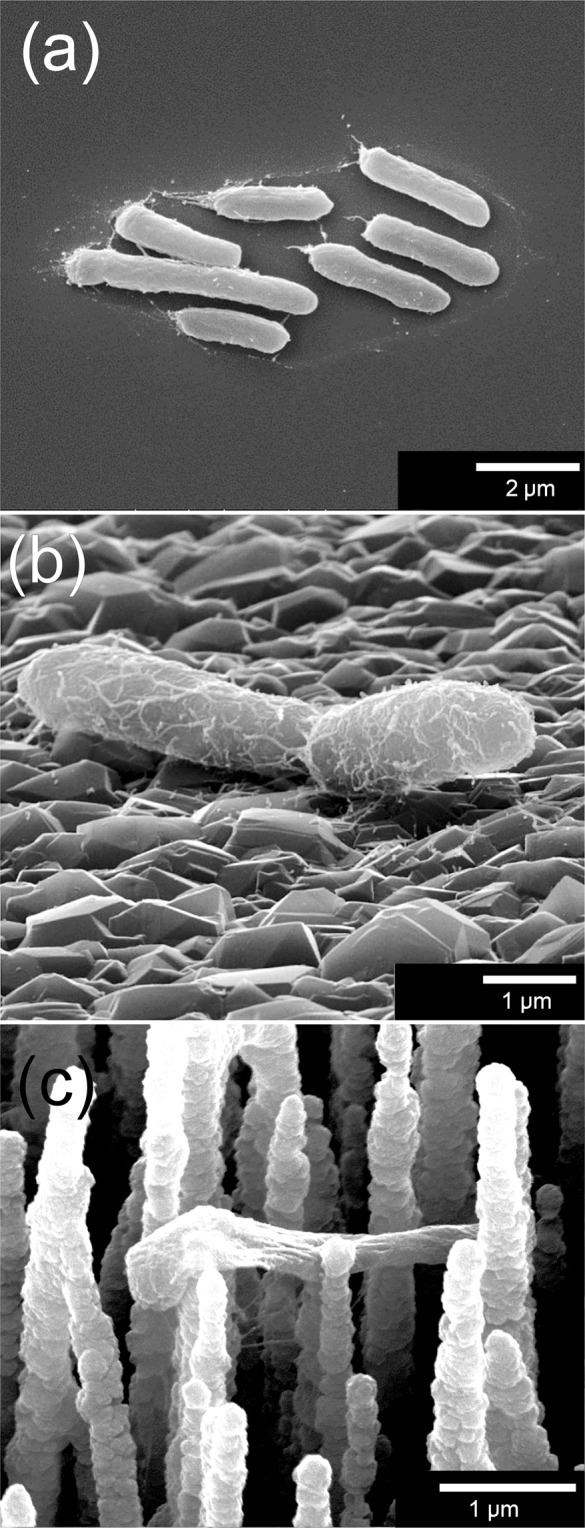
SEM images of *E. coli*, showing healthy, turgid bacterial cells on (**a**) flat Si and (**b**) flat diamond control samples. (**c**) A representative example of some of the bacteria on the H-terminated bD needle surface, which appear deformed and flaccid.

Surface-adhered *E. coli* were examined using SEM; the morphology of bacterial cells on a flat surface was compared to that on a bD surface. Figure 7(a),(b) illustrate that on both flat Si and diamond surfaces the cells appeared healthy – they were cylindrical in shape, turgid, and appeared anchored to the surfaces via appendages such as *fimbriae*. Conversely, cells in contact with bD needles (Fig. 7(c)) were deformed; they no-longer appeared as turgid bacilli but rather were stretched non-uniformly between a few points of contact.

**Figure 8:**
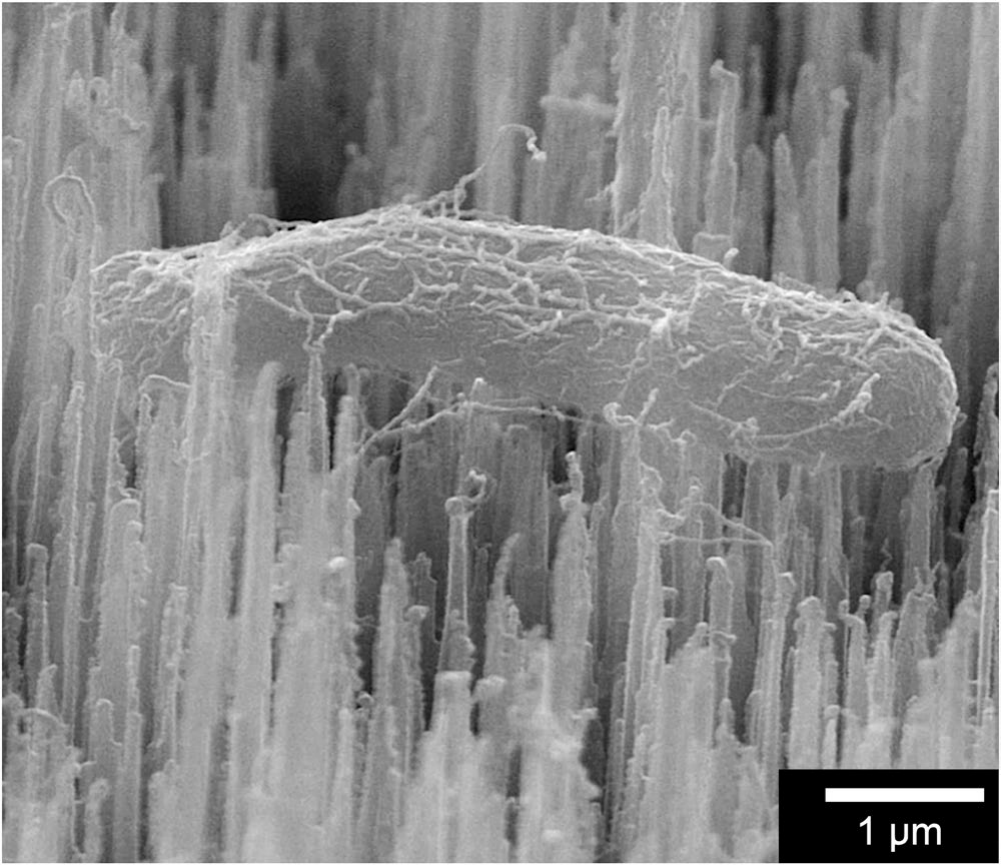
An example SEM image of an apparently healthy *E. coli* bacterium suspended between bSi spikes.

However, the story may be more complicated than the simple membrane-stretching model that bacterial death suggests. Figure 8 shows an SEM image of an apparently healthy bacterium, despite the cell seemingly lying on top of the spikey surface. In this case, it appears that the bacterial cell may be suspended *between* the bSi spikes, rather than sitting directly on the points. Here, the membrane is not being stretched by contact with the needle points, but rather bacterial adhesion to the surface is mediated via the *fimbriae* and other surface appendages. This might be evidence for why the superhydrophobic surfaces have a higher death rate than hydrophobic ones; the bacterial adhesins cannot attach strongly to a ‘non-stick’ surface.

## Conclusions

We have shown that nanostructured bD surfaces kill *E. coli* bacteria in significant numbers via a combination of mechanical effect, consistent with a membrane-stretching model of cell death, and chemical effects. Importantly, cell aggregation – the precursor to biofilm formation – was markedly reduced on the bD surfaces compared to that on flat control samples. The biocidal activity is mostly determined by the mechanical effect, however, the chemical nature of the surface did have an additional effect, with the number of bacteria being killed increasing roughly in line with the hydrophobicity of the surface. This was not due to prevention of the bacteria initially adhering, which was similar for all nanostructured surfaces, but was more likely a function of the weaker interaction of the bacterial surface appendages with the ‘non-stick’ surface. There are very few reports^22,23^ detailing the nature of these interactions, and so this is an important area for future investigation. Another potential area for future exploration is to chemically attach antibiotic molecules to the surface, via amide linkages to the NH_2_ terminations described above. This should greatly increase the chemical biocidal activity of the surface, which combined with the mechanical activity from the nanostructured surface, could make surfaces that exhibit very high antimicrobial activity.

These experiments were all done with the Gram-negative bacterium *E. coli*, which, due to its weak cell wall structure, might be expected to be susceptible to these surface modification strategies. Similar experiments with Gram-positive bacteria are also required if a more generally applicable anti-bacterial surface is to be developed.

Finally, we should be aware that for applications in the real world, bioresistant surfaces will realistically never be coated with exotic materials, such as bD. Instead, the nanostructured surfaces would be fabricated from more conventional materials like stainless steel, titanium, or polymers such as medical-grade rubber or PTFE. Nevertheless, the general findings from studies such as this one, optimising biocidal activity in terms of types, shapes and densities of surface morphology and chemical nature, will provide important information for the development of next-generation antibacterial surfaces made from more practical materials.

## Supplementary information

Supplementary Information

## References

[1] May PW (2000). Diamond thin films: A 21st century material. Philosophical Transactions of the Royal Society London A.

[2] Zanin H (2014). Porous boron-doped diamond/carbon nanotube electrodes. ACS Applied Materials and Interfaces.

[3] Yang N (2009). Vertically aligned diamond nanowires: Fabrication, characterisation and application for DNA sensing. Physica Status Solidi A.

[4] May PW (2016). Diamond coated ‘black-silicon’ as a promising material for high surface-area electrochemical electrodes and antibacterial surfaces. Journal of Materials Chemistry B.

[5] Oh J, Yuan C, Branz HM (2012). An 18.2%-efficient black-silicon solar cell achieved through control of carrier recombination in nanostructures. Nature Nanotechnology.

[6] Roy AB (2016). Black silicon solar cell: analysis optimization and evolution towards a thinner and flexible future. Nanotechnology.

[7] Kim W, Ng JK, Kunitake ME, Conklin BR, Yang J (2007). Interfacing silicon nanowires with mammalian cells. Journal of the American Chemical Society.

[8] Shalek AK (2010). Vertical silicon nanowires as a universal platform for delivering biomolecules into living cells. Proceedings of the National Academy of Sciences.

[9] Hazell G (2018). Studies of Black Silicon and Black Diamond as materials for antibacterial surfaces. Biomaterials Science.

[10] Ivanova EP (2013). Bactericidal activity of black silicon. Nature Communications.

[11] Al-Jumaili A, Alancherry S, Bazaka K, Jacob MV (2017). Review on the antimicrobial properties of carbon nanostructures. Materials.

[12] Medina O (2012). Bactericide and bacterial anti-adhesive properties of the nanocrystalline diamond surface. Diamond & Related Materials.

[13] Bandara CD (2017). Bactericidal effects of natural nanotopography of dragonfly wing on *Escherichia coli*. ACS Applied Materials and Interfaces.

[14] Wu S, Zuber F, Brugger J, Maniura-Weber K, Ren Q (2016). Antibacterial Au nanostructured surfaces. Nanoscale.

[15] Dickson MN, Liang EI, Rodriguez LA, Vollereaux N, Yee AF (2015). Nanopatterned polymer surfaces with bactericidal properties. Biointerphases.

[16] Linklater DP, Nguyen HKD, Bhadra CM, Juodkazis S, Ivanova EP (2017). Influence of nanoscale topology on bactericidal efficiency of black silicon surfaces. Nanotechnology.

[17] Kelleher SM (2015). Cicada wing surface topography: An investigation into the bactericidal properties of nanostructural features. ACS Applied Materials and Interfaces.

[18] Ivanova EP (2012). Natural bactericidal surfaces: Mechanical rupture of *Pseudomonas aeruginosa* cells by cicada wings. Small.

[19] Pogodin S (2013). Biophysical model of bacterial cell interactions with nanopatterned cicada wing surfaces. Biophysical Journal.

[20] Xue F, Liu J, Guo L, Zhang L, Li Q (2015). Theoretical study on the bactericidal nature of nanopatterned surfaces. Journal of Theoretical Biology.

[21] Li X (2016). Bactericidal mechanism of nanopatterned surfaces. Physical Chemistry Chemical Physics.

[22] Zhang X, Wang L, Levanen E (2013). Superhydrophobic surfaces for the reduction of bacterial adhesion. RSC Advances.

[23] Budil J (2018). Anti-adhesive properties of nanocrystalline diamond films against *Escherichia coli* bacterium: Influence of surface termination and cultivation medium. Diamond & Related Materials.

[24] Diu T (2014). Cicada-inspired cell-instructive nanopatterned arrays. Scientific Reports.

[25] Bereket W (2012). Update on bacterial nosocomial infections. European Review for Medical and Pharmacological Sciences.

[26] Donlan RM (2002). Biofilms: Microbial Life on. Surfaces, Emerging Infectious Disease.

[27] Luppens SB, Reij MW, van der Heijden RW, Rombouts FM, Abee T (2002). Development of a standard test to assess the resistance of *Staphylococcus aureus* biofilm cells to disinfectants. Applied Environmental Microbiology.

[28] Karlowsky JA, Kelly LJ, Thornsberry C, Jones ME, Sahm DF (2002). Trends in antimicrobial resistance among urinary tract infection isolates of *Escherichia coli* from female outpatients in the United States. Antimicrobial Agents and Chemotherapy.

[29] Huang CT (1995). Non-uniform spatial patterns of respiratory activity within biofilms during disinfection. Applied Environmental Microbiology.

[30] Davies D (2003). Understanding biofilm resistance to antibacterial agents. Nature Reviews Drug Discovery.

[31] Costerton JW, Stewart PS, Greenberg EP (1999). Bacterial biofilms: a common cause of persistent infections. Science.

[32] Beloin C, Roux A, Ghigo JM (2008). *Escherichia coli* biofilms. Current Topics in Microbiology and Immunology.

[33] Oxford Instruments Plasma Technology, Ltd website, https://www.oxford-instruments.com/businesses/nanotechnology/plasma-technology.

[34] Fox OLJ, Holloway JOP, Fuge GM, May PW, Ashfold MNR (2010). Electrospray deposition of diamond nanoparticle nucleation layers for subsequent CVD diamond growth. Materials Research Society Symposium Proceedings.

[35] Al-Jeda, T. *Construction and Operation of a Plasma System to Fluorinate and Aminate the Surfaces of Diamond Thin Films*, MSci thesis, University of Bristol, UK, (available on request from the corresponding author) (2018).

[36] Salvadori MC (2010). Termination of diamond surfaces with hydrogen, oxygen and fluorine using a small, simple plasma gun. Diamond & Related Materials.

