# Supplementary material for: Studies of Black Diamond as an antibacterial surface for Gram Negative bacteria: the interplay between chemical and mechanical bactericidal activity
Source: Sci Rep. 2019 Jun 19;9:8815. doi: 10.1038/s41598-019-45280-2 (PMC6584650; doi:10.1038/s41598-019-45280-2)
Supplement: Supplementary file 1 — Supplementary Information [file 41598_2019_45280_MOESM1_ESM.docx]

# Studies of Black Diamond as an antibacterial surface for Gram Negative bacteria: the interplay between chemical and mechanical bactericidal activity

O. Dunseath, E. J. W. Smith, T. Al-Jeda, J. A. Smith, S. King, P. W. May, A. H. Nobbs, G. Hazell, C. C. Welch, B. Su

Supplementary Information

Fig.S1. Laser Raman spectrum (514 nm excitation) of the bD needles. Key: ‘Si’ is the second-order peak from Si which remains embedded within the needles; ‘ND’ is a feature associated with the sp^2^ carbon located in the grain boundaries of nanodiamond material, sometimes called *trans*-polyacetylene; ‘Dia’ is the characteristic diamond peak at 1332 cm^-1^, which is broadened here due to the small grain-size of the crystallites, ‘D’ and ‘G’ are the disordered and ordered graphite peaks, respectively, arising from the sp^2^ carbon species at the grain boundaries.

Ref: J. Filik. Raman Spectroscopy: a simple, non-destructive way to characterise diamond and diamond-like materials. *Spectroscopy Europe*, **17**, 10-17 (2005).
